# Antioxidant and inflammatory aspects of lipoprotein-associated phospholipase A_2 _(Lp-PLA_2 _): a review

**DOI:** 10.1186/1476-511X-10-170

**Published:** 2011-09-28

**Authors:** Isis T Silva, Ana PQ Mello, Nágila RT Damasceno

**Affiliations:** 1 Departamento de Nutrição, Faculdade de Saúde Pública, Universidade de São Paulo, São Paulo, SP, Brasil

**Keywords:** Lp-PLA_2 _, Cardiovascular risk, antioxidant, pro-inflammatory

## Abstract

The association of cardiovascular events with Lp-PLA_2 _has been studied continuously today. The enzyme has been strongly associated with several cardiovascular risk markers and events. Its discovery was directly related to the hydrolysis of the platelet-activating factor and oxidized phospholipids, which are considered protective functions. However, the hydrolysis of bioactive lipids generates lysophospholipids, compounds that have a pro-inflammatory function. Therefore, the evaluation of the distribution of Lp-PLA_2 _in the lipid fractions emphasized the dual role of the enzyme in the inflammatory process, since the HDL-Lp-PLA_2 _enzyme contributes to the reduction of atherosclerosis, while LDL-Lp-PLA_2 _stimulates this process. Recently, it has been verified that diet components and drugs can influence the enzyme activity and concentration. Thus, the effects of these treatments on Lp-PLA_2 _may represent a new kind of prevention of cardiovascular disease. Therefore, the association of the enzyme with the traditional assessment of cardiovascular risk may help to predict more accurately these diseases.

## 1. Introduction

The physiopathology of cardiovascular disease (CVD) is marked by the presence of atherosclerosis that involves endothelial dysfunction, inflammation, oxidative stress, insulin resistance and dyslipidemia.

Even considering the early diagnosis and the new variety of treatments for CVD, the American College of Cardiology still predicts that there will be 25 million cases only in USA until the end of 2050 [1]. Furthermore, given the current importance of CVD, thanks to its high worldwide prevalence that accounts for nearly 30% of the global deaths [2], the monitoring of the new biomarkers and risk factors represents an important focus of new researches.

In this context, lipoprotein-associated phospholipase A_2 _(Lp-PLA_2 _) represents a potential cardiovascular risk marker, given its correlations with coronary disease and stroke [3-7]. Initially, Lp-PLA_2 _was recognized by its action on hydrolyzing platelet-activating factor (PAF); such characteristic has assigned to it the first name platelet-activating factor acetylhydrolase (PAF-AH) [8].

Despite the other important reviews of Lp-PLA_2 _[9-11], the question of whether high activity of Lp-PLA_2 _is a causal event or a result of atherosclerosis remains open. Therefore, the main goal of this review is to show the antioxidant and inflammatory role of Lp-PLA_2 _and its connection with atherosclerosis, aiming to contribute to the discussions of atherogenic or anti-atherogenic role of Lp-PLA_2 _. We also discuss possible mechanisms of modulation of Lp-PLA_2 _.

## 2. Biochemistry and structural aspects

**Figure 1:**
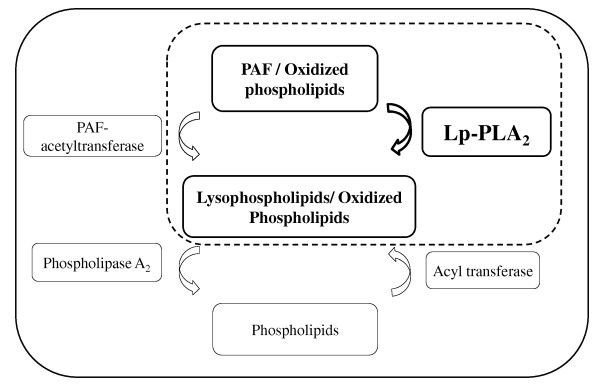
**Role of Lp-PLA_2 _on the generation of lysophospholipids**.

A brief biological background is necessary to comprehend mechanisms enrolling Lp-PLA_2 _and atherosclerosis. Platelet-activating factor (PAF) is an active phospholipid related to many pathologic and physiologic reactions [12]. The PAF is formed through two reactions (Figure 1). Firstly, the cytosolic phospholipase A_2 _(cPLA_2 _) acts on membrane phospholipids producing lysophospholipids; then, the lysophospholipids are modified by PAF acetyltransferase, resulting in the formation of PAF [13]. Thus, PAF concentration is modulated by Lp-PLA_2 _activity [13,14].

Lp-PLA_2 _was discovered on 1980 and it was classified as a Ca^2+^-independent PLA_2 _[8], produced by a wide range of inflammatory and non-inflammatory cells [15-17]. It is considered a member of phospholipases family (PLA_2 _), although exhibits different properties when compared to other PLA_2 _[18]. In addition, while Lp-PLA_2 _is specific for the breakdown of PAF and oxidized fatty acid residues, PLA_2 _is specific for phospholipids containing two long chain acyl groups [18-21].

Another feature of Lp-PLA_2 _is that it shows different isoforms, though the more common types are distributed in intracellular [22] and extracellular compartment [8]. Intracellular Lp-PLA_2 _shows two variables, I and II [23], while brain tissue exhibits a subtype named Lp-PLA_2 _-Ib [24]. The Lp-PLA_2 _type II consists of a 40-KDa polypeptide chain, and has been associated with antioxidant properties [25]. The extracellular Lp-PLA_2 _, identified as plasma form, circulates in association primarily with LDL (80-85%) and on minor portion with HDL (15-20%), having its activity strongly correlated with the cholesterol concentrations [26,27]. Lp-PLA_2 _has been extracted from human plasma and erythrocytes, bovine brain, liver and seminal plasma, guinea pig peritoneal fluid and plasma, mouse plasma and platelets, cultured rat Kupffer cell- and hepatocyte-conditioned media, rat bile and the parasite *Nippostrongylus brasiliensis *[28]. On the same hand, it was verified that the different isoforms of Lp-PLA_2 _define distinct activities for the enzyme [23,29,30].

## 3. Antioxidant role of Lp-PLA_2 _

The oxidative stress is closely associated with inflammation and bioactive lipid formation. These bioactive lipids, such as PAF, PAF-like substances, and oxidized phospholipids, have been identified in atherosclerotic plaque [31]. PAF-like products are formed when the phospholipids of the cellular membrane suffers oxidative damage, resulting in compounds that have structures with shorter peroxidized residues at their second carbon and that mimic the action of PAF [32].

In presence of oxidized phospholipids, Lp-PLA_2 _removes these fragments acting as an antioxidant. Matsuzawa *et al. *[33], suggested that the over expression of Lp-PLA_2 _protects the cells of reactive oxygen species (ROS)-induced apoptosis through oxidized phospholipids hydrolysis.

In addition, oxidized LDL and LDL(-) are known to be important factors on the atherosclerosis initiation and development [34-36]. Heery *et al. *[37] demonstrated that the formation of oxidized phospholipids in LDL stimulates Lp-PLA_2 _activity. It is most likely that the Lp-PLA_2 _hydrolysis of the lipids present in this particle represents an important antiatherogenic role. In this context, Watson *et al. *[38] showed that the Lp-PLA_2 _, hydrolyzing oxidized phospholipids, minimizes the generation of highly oxidized LDL, increasing the minimally oxidized LDL content. Subsequently, Benitez *et al. *[39] found that the major portion of Lp-PLA_2 _was associated with LDL(-) in detriment to LDL(+), suggesting that the release of chemotactic induced by LDL(-) could be a consequence of the high Lp-PLA_2 _activity. Indeed, LDL(-) can be generated by Lp-PLA_2 _, although the origin of this sub-group of LDL could to be also compatible with oxidative reaction and other mechanisms such as non enzymatic glycosylation, changes on Apo E (apolipoprotein E) and Apo CII (apolipoprotein CII), non esterified fat acids (NEFAS) enrichment or cross linking with hemoglobin [40].

Lourida *et al. *[41] showed that Lp-PLA_2 _activity is important for reducing the immunogenicity of oxLDL, a phenomenon that can be attributed to the decreasing of oxidized phospholipids in patients with coronary artery disease and healthy ones. More recently, Noto *et al. *[42] showed in animals that Lp-PLA_2 _protects lipoproteins from oxidation, producing less proatherogenic lipoproteins and preserving HDL functions. In this direction, Bazan [43] proposed that recombinant Lp-PLA_2 _could be a potential tool directed to antiatherogenic therapy.

## 4. Inflammatory action of Lp-PLA_2_

Despite the antioxidant potential described above, the association of Lp-PLA_2 _with inflammatory reactions represents the majority of the studies in literature in the last years.

When Lp-PLA_2 _hydrolyzes bioactive lipids, reducing their biological activity, the most generated metabolites are the lysophospholipids. These lipids are involved with atherosclerotic process and show a deleterious role of Lp-PLA_2 _, contributing to the inflammatory response against oxidized lipoproteins [39,44,45]. These compounds generated by phospholipases A_2 _during cell activation, injury, or apoptosis, are known to affect the function of neutrophils and of a diversity of cell types [46], and can be also produced by phospholipase A1 and by the action of lecithin-cholesterol acyltransferase (LCAT) or endothelial lipase. There are many different lysophospholipids, but the main product of Lp-PLA_2 _action is lysophosphatidylcholine [47]; these metabolic processes occur in physiological conditions.

Furthermore, lysophospholipids from apoptotic cells contribute to attract monocytic cells and primary macrophages [48,49]. In this context, Steinbrecher & Pritchard [45] showed that oxLDL, on the presence of phenylmethanesulphonylfluoride (PMSF), an inhibitor of Lp-PLA_2 _, has lower values of lysophospholipids. In this fashion, Muller *et al. *[50] proposed that lysophosphatidylcholine represents a biomarker of the intensity of the reactive oxygen species production at the inflammatory site. Accordingly, Lavi *et al. *[51] found that patients with early coronary atherosclerosis had higher lysophosphatidylcholine when compared with control subjects. This profile was confirmed by Herrmann *et al. *[52], who showed that carotid artery plaques of patients with cardiac events presented higher Lp-PLA_2 _, lysophospholipids, macrophage and collagen content when compared to patients without events.

Studying the effects of oxLDL, Kuniyasu *et al. *[53] demonstrated that oxLDL, and particularly, the lysophosphatidylcholine present in this particle, enhances the plasminogen activator inhibitor-1 expression. Vickers *et al. *[54] demonstrated also that lysophosphatidylcholine can contribute to calcify vascular cells on the atherosclerotic plaque, through up-regulation of osteogenic genes and proteins. Hence, many events present in atherosclerotic process involve directly Lp-PLA_2 _or its products.

**Figure 2:**
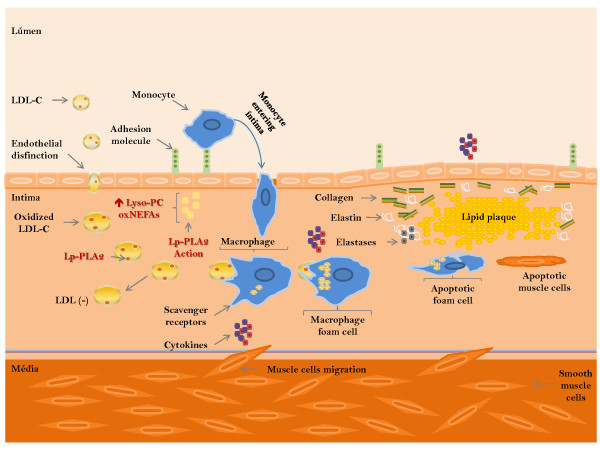
**Possible actions of Lp-PLA_2 _in the atherosclerotic process**.

Figure 2 summarizes the possible atherogenic mechanisms involving Lp-PLA_2 _. In this context, there can be an individual with dyslipidemia, obesity, hypertension, insulin resistance and oxidative stress, and therefore, highly prone to atherosclerosis. These factors contribute initially to the endothelial dysfunction, characterized by the expression of more adhesion molecules and by larger spaces between endothelial cells. Thus, the LDL, macrophages and T lymphocytes can transmigrate more easily to arterial intima. This LDL particle shows a phenotype more atherogenic, being dense and small, characteristics that make it more susceptible to oxidation. In this site, the reduced content of antioxidants favors the high production of free radicals, and consequently oxidative modifications of LDL. Thus, the Lp-PLA_2 _will be activated by oxidized phospholipids present in OxLDL.

The enzyme minimizes modifications of OxLDL, hydrolyzing its oxidized phospholipids; this may be interpreted as an antioxidant action. However, during this process, there are produced high contents of lysophospholipids and oxidized non esterified fat acids (OxNEFAS) that promote adhesion molecules expression and attract macrophages to the arterial intima. The OxLDL, lysophospholipids and OxNEFAS also stimulates cytokines production, like TNF-α and IL-6, which increase the inflammatory profile in the region of the plate. The activated macrophages, through scavenger receptors, phagocyte OxLDL, gradually turning up in foam cells. The muscle cells are also attracted, and migrate to the intima, where they produce collagen, elastin and elastases, involving and stabilizing the lipid plaque. Subsequently, the macrophages become apoptotic, as well as the muscle cells, causing released of lipids in the plaque. In this process, the presence of OxLDL, as well as lysophospholipids and OxNEFAS produced by Lp-PLA_2_, is always stimulating the growth of the plaque; these are factors that can be decisive to plaque rupture susceptibility that can culminate in a cardiovascular event.

## 5. Lp-PLA_2 _and Cardiometabolic Risk

**Figure 3:**
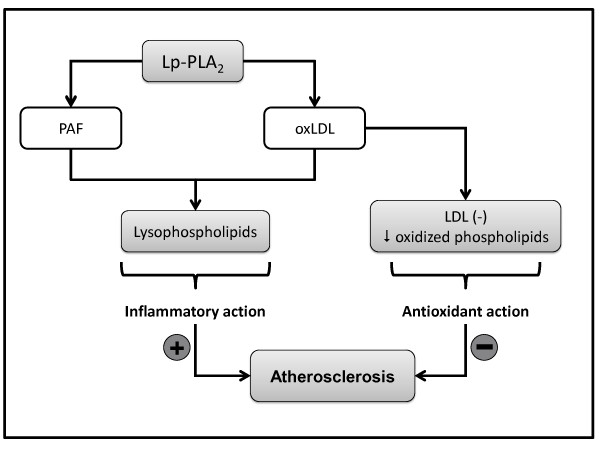
**Lp-PLA_2 _action on cardiovascular disease**.

Taking into account the mechanisms described, Lp-PLA_2 _could influence the cardiometabolic risk; the Figure 3 expresses the two main possibilities for action of the enzyme in cardiovascular disease context; the antioxidant action, where these hydrolysis reduce the oxidized phospholipids in plasma and the oxLDL contributing to generation of LDL(-); the inflammatory action, where the hydrolysis of PAF, PAF-like products or oxidized phospholipids generate lysophospholipids that stimulate inflammation, so the atherogenic process is stimulated.

According to Campo *et al. *[55], Lp-PLA_2 _activity was significantly associated with LDL-cholesterol in hypercholesterolemic patients. As a matter of fact, dyslipidemia promotes an increase in plasma Lp-PLA_2 _activity and alters the enzyme distribution between apo B- and apo AI-containing lipoproteins, as observed by Tsimihodimos *et al. *[56]. The role of LDL-associated Lp-PLA_2 _remains controversial, possibly because of the difficulty in analyzing the actions of the enzyme in the dense LDL particle [57].

On the other hand, studies also have showed that the enzyme activity associated with HDL particles can play an antiatherogenic action. Theilmeier *et al. *[58] demonstrated by *in vitro *and *in vivo *models that HDL-Lp-PLA_2 _(HDL-Lipoprotein-associated phospholipase A_2 _) activity was linked to reduction of endothelial adhesiveness and of macrophage recruitment to lesion prone sites. Afterwards, the same group demonstrated that atorvastatin induced the increase of HDL-Lp-PLA_2 _activity and the reduction of LDL-Lp-PLA_2 _(LDL-Lipoprotein-associated phospholipase A_2 _) activity [56].

Papavasiliou *et al. *[59], investigating chronic kidney disease patients, found an increase in plasma Lp-PLA_2 _activity and a reduction of the ratio of HDL-Lp-PLA_2 _to plasma when compared to controls. In the same way, Rizos *et al. *[26] demonstrated that patients with metabolic syndrome have higher Lp-PLA_2 _activity than controls. Nevertheless, the Lp-PLA_2 _content in HDL was lower; these results were confirmed by Lagos *et al. *[60], who observed that the HDL-Lp-PLA_2 _activity was lower in patients with metabolic syndrome. Okamura *et al. *[31] suggested that even the Lp-PLA_2 _having an important function in atherogenesis, its association with HDL plays the opposite role, as observed by high LDL-Lp-PLA_2 _to HDL-Lp-PLA_2 _ratio in patients with atrial fibrillation.

In this fashion, Noto *et al. *[61] showed that diabetic patients with metabolic syndrome have significantly higher Lp-PLA_2 _activity than those without this disease, reflecting its importance to metabolic risk. Following up, in a cohort with 299 subjects, Allison *et al. *[62] demonstrated that an increment of one standard deviation in Lp-PLA_2 _activity was associated with a higher risk of CVD in five years, but not with mortality. Kiechl *et al. *[63], in a prospective study, demonstrated that oxidized phospholipids/apo B ratio predicted the cardiovascular risk, being the Lp-PLA_2 _activity an amplifier of this risk.

Accordingly, Sabatine *et al. *[4] observed that an elevated level of Lp-PLA_2 _is a predictor of adverse cardiovascular outcomes, independently of the traditional clinical risk factors in patients with stable coronary artery disease. Persson *et al. *[64] observed that this enzyme was strongly correlated with lipid fractions and the degree of carotid artery atherosclerosis; this study showed that the association with cardiovascular risk is stronger for activity than for mass, reinforcing the impact of activity in atherogenesis [64].

In a prospective population-based survey, which occurred between 1990 and 2005, it was verified that Lp-PLA_2 _was higher in subjects with incidence of CVD [5]. In the same year, Jenny *et al. *[65] showed that subjects with heart failure have the elevation of Lp-PLA_2 _levels associated with an increase in the mortality risk. It was detected also that subjects aged > 65 years presented an association between the Lp-PLA_2 _and myocardial infarction [65]. An increasing risk of major adverse cardiac events associated with elevated Lp-PLA_2 _was also observed in community-based cohort of patients with acute coronary syndrome [6].

More recently, the Lp-PLA_2 _Studies Collaboration, analyzing 32 prospective studies, confirmed that the enzyme activity and mass were related to proatherogenic lipids and vascular risk [66]. The study showed also that the association of the enzyme activity with lipid markers is stronger than the association with mass [66]. Recently, the authors of this review verified that the Lp-PLA_2 _activity in adolescents is positively associated with total cholesterol, LDL-C, insulin, glucose, HOMA-IR, Apo B (apolipoprotein B)/Apo AI (apolipoprotein AI) ratio and negatively related to HDL size.

In contrast with the studies above, Tsironis *et al. *[67] showed that patients with coronary disease exhibit reduced LDL-Lp-PLA_2 _mass and catalytic efficiency, suggesting a diminished ability to degrade pro-inflammatory phospholipids.

Therefore, it is probably that Lp-PLA_2 _shows a dual action, directly dependent on its association with LDL (proatherogenic) or HDL (antiatherogenic). Table 1 summarizes the antioxidant, inflammatory and neutral links between Lp-PLA_2 _and cardiometabolic risk.

**Table 1:** Potential action of the Lp-PLA_2 _, according to studies with distinct design.

Study design	Action	Reference
Experimental	Cells ROS protection.	[33]
Experimental	↓ bioactivity phospholipids in oxLDL	[37]
Experimental	↓ oxidized phospholipids in mildly oxLDL	[38]
Case/Control	≈ Oxidized phospholipids and anti-Lp-PLA_2 _	[41]
Case/Control	↓ HDL oxidation, foam cell and autoantibodies titers.	[42]
Case/Control	↓ HDL-Lp-PLA_2 _activity	[25]
Case/Control	↓ HDL-Lp-PLA_2 _activity	[60]
Case/Control	↑ LDL-Lp-PLA_2 _to HDL-Lp-PLA_2 _ratio	[31]
Case/Control	↓ HDL-Lp-PLA_2 _and ↑ of LDL-Lp-PLA_2 _	[56]
Case/Control	≈ Lp-PLA_2 _activity	[55]
Case/Control	↑ Lp-PLA_2 _activity	[61]
Cohort	↑ Lp-PLA_2 _activity in CHD mortality	[62]
Case/Control	↑ Lp-PLA_2 _activity	[63]
Cohort	Predictor of cardiovascular outcomes	[4]
Cohort	Lp-PLA_2 _correlated with cardiovascular risk factors	[64]
Cohort	Lp-PLA_2 _activity associated with MS and CVD	[5]
Cohort	Lp-PLA_2 _mass and activity associated with CVD	[65]
Cohort	↑ Lp-PLA_2 _activity associated with CVD	[6]
Meta-analysis	Lp-PLA_2 _mass and activity associated vascular risk	[66]

CVD: Cardiovascular Disease; CHD: Coronary Heart Disease; PAD: Peripheral Arterial Disease; ROS: Reactive Oxygen Species, MS: Metabolic Syndrome

## 6. Modulation of Lp-PLA_2_

Studies focused on Lp-PLA_2 _modulation are little explored in literature, despite of its possible manipulation. Regarding that Lp-PLA_2 _is associated with cholesterol and oxidized lipids in LDL and HDL, it is probable that drugs and environment factors, capable of modulating the lipid metabolism, may change the mass and the activity of this enzyme.

Gerra *et al. *[68] showed that lovastatin was responsible for the simultaneous decrease of LDL-C level and Lp-PLA_2 _activity. Similary, Tsimihodimos *et al. *[56] found reduced Lp-PLA_2 _activity in plasma of hypercholesterolemic patients under atorvastatin therapy, with a reduction in LDL-Lp-PLA_2 _activity; in contrast, there was no modification in HDL-Lp-PLA_2 _activity. The same authors, in an investigation of the effect of fenofibrate on hypercholesterolemic patients, observed a reduction in the LDL-Lp-PLA_2 _activity and an increase of the HDL-Lp-PLA_2 _activity [69]. Schaefer *et al. *[70], comparing the effect of atorvastatin with placebo in coronary heart disease patients observed a reduction of Lp-PLA_2 _under therapy.

Studying the effect of cholesterol feeding and simvastatin treatment on rabbits, Zhang *et al. *[71] found that the LDL-Lp-PLA_2 _activity increased with cholesterol feeding and decreased after the treatment. In this way, O'Donoghue *et al. *[72] found that an intensive statin therapy was responsible for 20% of reduction in LDL-Lp-PLA_2 _, in average. Likewise, Schaefer *et al. *[70] observed that simvastatin determined a reduction of the Lp-PLA_2 _mass in 26%.

In the same way, atorvastatin or fenofibrate therapies can increase the ratio of HDL-Lp-PLA_2 _to plasma Lp-PLA_2 _(or to LDL-Lp-PLA_2 _) [57]. Also, the effect of gemfibrozil was monitored in men with low HDL-C, and it was verified that individuals in highest quartile of Lp-PLA_2 _showed reduction of cardiovascular events [73]. The use of darapladib (oral Lp-PLA_2 _inhibitor) by coronary patients caused a reduction of 59% of the enzyme activity after 12 months of treatment; concomitantly, the placebo group presented a significant increase of necrotic core volume when compared to the therapy group [74]. In a complementary study, the combined effect of atorvastatin and darapladib was evaluated in patients with coronary heart disease in the course of 12 weeks; the individuals under darapladib showed a reduction of approximately 54% in the Lp-PLA_2 _activity when compared with controls [75].

Investigating patients under low-fat-diet and orlistat treatment, fenofibrate or both drugs during six months, Filippatos *et al. *[76] observed a significant reduction of Lp-PLA_2 _activity in all groups (14%, 22% and 35%, respectively) when compared to basal time. The results suggested the combination of the two treatments as the optimal therapy.

Hence, the direct influence of lipid metabolism on Lp-PLA_2 _was confirmed by the efficiency of hypocholesterolemic drugs. Nonetheless, a similar profile was not observed in patients under anti-hypertension treatment: Spirou *et al. *[77] and Rizos *et al. *[78] verified that anti-hypertensive was not able to change Lp-PLA_2 _activity.

Despite the positive effect on Lp-PLA_2 _demonstrated by application of drugs, many studies have also investigated the influence of diet and other environment factors on the enzyme. In this context, Pedersen *et al. *[79] compared the effects of high (6.6 g), low (2.0 g) and control doses of n-3 polyunsaturated fatty acids in some metabolic parameters; they did not observe any effect on Lp-PLA_2 _activity. Recently, in a sub-sample (n = 150, follow up = 1 y) of PREDIMED study, the authors of the present work, comparing diets enhanced with a mix of nuts (30 g/d), olive oil (50 g/d) or with low concentration of saturated fat (< 7%), observed a reduction in Lp-PLA_2 _only in the nuts group [NRTD, personal communication].

The effect of selenium on Lp-PLA_2 _was recently evaluated [80] on rats, subject to three different diets (control, high fat and high fat enhanced with selenium). The results showed that the Lp-PLA_2 _levels in control group were lower than the other groups, and that the selenium did not affect this enzyme.

The Nurses' Health Study demonstrated that the replacement of energy from carbohydrates for proteins, as well as the alcohol consumption or use of cholesterol-lowering drugs, were associated with a reduction in the Lp-PLA_2 _activity. Smoking, overweight, aspirin use, hypercholesterolemia and age were, nevertheless, related to the elevation of Lp-PLA_2 _activity [81]. In addition, obese and non-diabetic women submitted to a weight reduction program showed a significant reduction in Lp-PLA_2 _activity, directly associated with VLDL-C [82]. The influence of the nutritional status on Lp-PLA_2 _activity was also evaluated in adolescents where it was positively associated with body mass index, waist circumference and fat mass percentage [83].

Finally, Chen *et al. *[84] compared vegetarians with omnivores and observed that vegetarians presented lower Lp-PLA_2 _activity, with lower total cholesterol and LDL-cholesterol, but with increased chances of higher C-reactive protein.

## 7. Conclusion

Initially, the discovery of the enzyme Lp-PLA_2 _was associated with its ability to hydrolyze PAF and phospholipids, what was seen as a protective function. The enzyme acts as an antioxidant in the presence of oxidized phospholipids. Thus, Lp-PLA_2 _, in this sense, represents an important factor, reducing the oxLDL atherogenicity. Nowadays, however, its association with cardiovascular events is the most outstanding characteristic observed.

In addition, associations with several cardiovascular risk markers were also described in the literature. The enzyme hydrolyzes bioactive lipids, reducing their biological activity; the major metabolites generated in the process are the lysophospholipids. Given these results, the enzyme has been associated with a pro-inflammatory action, explained mainly by the production of these compounds that stimulate the inflammatory process in the region of the atherosclerotic plate.

Focusing on the enzyme antiatherogenic function, several studies have been evaluating the distribution of Lp-PLA_2 _in the lipid fractions. Surprisingly, the HDL-Lp-PLA_2 _enzyme has proven beneficial results to the atherosclerotic process. In the same sense, LDL-Lp-PLA_2 _is linked to higher cardiovascular risk. Drugs and diet components that alter the lipid profile, the insulin resistance and the inflammatory markers also affect the enzyme activity and its concentration. Possibly, the effects of these components on the Lp-PLA_2 _activity, according to the lipid fraction, represent a new kind of prevention of CVD.

The traditional assessment of cardiovascular risk is based on lipid profile, inflammation and body composition. Since the control of these variables seeks to reduce cardiovascular events and this enzyme is strongly related to them, it is probable that the monitoring of its activity and its distribution on lipoproteins will predict better the cardiovascular risk.

## List of abbreviations

Apo AI: Apolipoprotein AI; Apo B: Apolipoprotein B; Apo CII: Apolipoprotein CII; Apo E: Apolipoprotein E; cPLA_2 _: Cytosolic phospholipase A_2 _; CVD: Cardiovascular disease; HDL-C: High density lipoprotein cholesterol; HDL-Lp-PLA_2 _: HDL-Lipoprotein-associated phospholipase A_2 _; LCAT: Lecithin-cholesterol acyltransferase; LDL(-): Electronegative low-density lipoprotein; LDL-C: Low density lipoprotein cholesterol; LDL-Lp-PLA_2 _: LDL-Lipoprotein-associated phospholipase A_2; _Lp-PLA_2 _: Lipoprotein-associated phospholipase A_2 _; NEFAs: Non esterified fat acids; oxLDL: Oxidized low-density lipoprotein; OxNEFAS: Oxidized non esterified fat acids; PAF: Platelet-activating factor; PAF-AH: Platelet-activating factor acetylhydrolase; PAF-like: Platelet-activating factor like; PLA_2 _: Phospholipases family; PMSF: Phenylmethanesulphonylfluoride; ROS: Reactive oxygen species; VLDL-C: Very low density lipoprotein cholesterol.

## Competing interests

The authors declare that they have no competing interests.

## Authors' contributions

ITS wrote the manuscript, APQM reviewed the manuscript and NRTD designed, drafted and critically reviewed the manuscript. All authors approved the final version of the manuscript.
